# P57 Comparative analysis of antibiotic susceptibility in *Escherichia coli* using the agar disc diffusion method

**DOI:** 10.1093/jacamr/dlag102.063

**Published:** 2026-06-26

**Authors:** Hoor Mohamed, Erdi Dilek

**Affiliations:** University of Hertfordshire, Hatfield AL10 9AB, UK; University of Hertfordshire, Hatfield AL10 9AB, UK

## Abstract

**Background:**

Antimicrobial resistance (AMR) is widely recognized as one of the most significant threats to modern medicine, contributing to approximately 4.95 million deaths annually.^1^. A key contributor to this burden is *Escherichia coli*, the leading cause of bacteraemia in England, with the highest incidence of bloodstream infections, surpassing both *Staphylococcus aureus* and *Klebsiella pneumoniae*.^2^ As resistance rates continue to rise, the need for accurate antimicrobial susceptibility profiling is increasingly critical. Agar disc diffusion provides a standardized and reproducible method for generating comparative data across antibiotic classes, supporting evidence-based antimicrobial stewardship.

**Objectives:**

To measure and compare zones of inhibition produced by four antibiotics, tetracycline, ampicillin, streptomycin, and penicillin, against *Escherichia coli* K-12 using the agar disc diffusion method across three repeated weekly experimental timepoints.

**Methods:**

*Escherichia coli* K-12 was cultured on nutrient agar plates using strict aseptic technique, with a uniform bacterial lawn established by sterile swab inoculation. Four antibiotic discs tetracycline, ampicillin, streptomycin, and penicillin were applied using sterile forceps and plates incubated at 37°C for 24 h. The experiment was replicated across three consecutive weekly timepoints to evaluate reproducibility. Zones of inhibition were visually assessed and systematically recorded following each incubation period. Mean zone diameters and standard deviations were calculated across all three replicates. All variables, including bacterial strain, disc concentrations, agar type, and incubation conditions, were standardized throughout to ensure experimental rigour.

**Results:**

Penicillin and ampicillin produced no zones of inhibition in any of the three weeks, meaning *E. coli* K-12 was fully resistant to both antibiotics. This was expected, as *E. coli* is a Gram-negative bacterium with an outer membrane that blocks these antibiotics from entering the cell. Tetracycline produced clear zones of inhibition in all three weeks 2.5 cm in Week 1, 2.7 cm in Week 2, and 2.8 cm in Week 3 giving a mean of 2.67 cm. The standard deviation was 0.15 cm, meaning results were very consistent across weeks. Streptomycin also produced visible zones 2.6 cm, 2.9 cm, and 2.4 cm with a mean of 2.63 cm, but a higher standard deviation of 0.25 cm, showing more variation between weeks. Both tetracycline and streptomycin were classified as susceptible, as all measurements exceeded the 1.5 cm threshold.

**Conclusions:**

This study found that *E. coli* K-12 responded differently to each antibiotic tested. Penicillin and ampicillin showed no inhibitory effect, because *E. coli* possesses an outer membrane that prevent these antibiotics from entering the cell, and produces β-lactamase enzymes that break them down. This highlights why identifying the bacterial species before selecting an antibiotic is critically important. Tetracycline showed partial effectiveness with consistent results, while streptomycin was less reliable across the three weeks. These findings underline the importance of pathogen-specific susceptibility testing in guiding appropriate antibiotic selection and supporting antimicrobial stewardship. Future studies incorporating CFU counting would provide valuable quantitative validation of these results.
